# Ligand-induced stabilization of the aptamer terminal helix in the *add* adenine riboswitch

**DOI:** 10.1261/rna.040493.113

**Published:** 2013-11

**Authors:** Francesco Di Palma, Francesco Colizzi, Giovanni Bussi

**Affiliations:** SISSA-Scuola Internazionale Superiore di Studi Avanzati, 34136 Trieste, Italy

**Keywords:** P1 stem, RNA aptamer, adenine riboswitch, molecular dynamics simulation, free energy calculation

## Abstract

Riboswitches are structured mRNA elements that modulate gene expression. They undergo conformational changes triggered by highly specific interactions with sensed metabolites. Among the structural rearrangements engaged by riboswitches, the forming and melting of the aptamer terminal helix, the so-called P1 stem, is essential for genetic control. The structural mechanisms by which this conformational change is modulated upon ligand binding mostly remain to be elucidated. Here, we used pulling molecular dynamics simulations to study the thermodynamics of the P1 stem in the *add* adenine riboswitch. The P1 ligand-dependent stabilization was quantified in terms of free energy and compared with thermodynamic data. This comparison suggests a model for the aptamer folding in which direct P1-ligand interactions play a minor role on the conformational switch when compared with those related to the ligand-induced aptamer preorganization.

## Abstract (precis)

All-atom pulling molecular dynamics simulations of the adenine-sensing *add* A-riboswitch to characterize the ligand influence on the forming and melting of the aptamer terminal helix (P1). The P1 ligand-dependent stabilization was quantified in terms of free energy and compared with thermodynamic data. This comparison suggests a model for the aptamer folding in which direct P1-ligand interactions play a minor role on the conformational switch when compared with those related to the ligand-induced aptamer preorganization.

## INTRODUCTION

**FIGURE 1.**
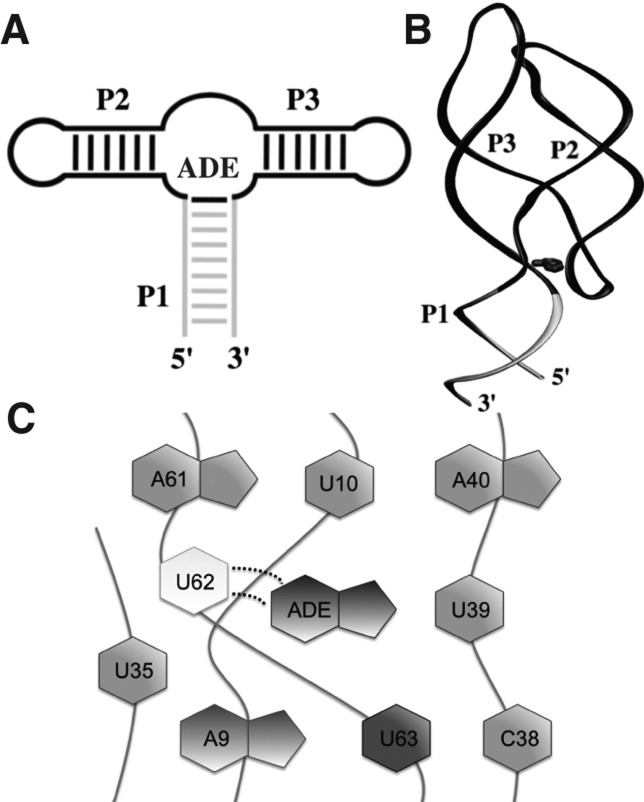
Adenine riboswitch aptamer and binding site. (*A*) Secondary structure elements and (*B*) 3-dimensional structure with bound adenine. The P1 stem is gray; the other stems and loops are black. (*C*) Cartoon representation of the binding site; the two dotted lines represent the hydrogen bonds of the WC pairing between the U62 and the ligand.

Riboswitches are ligand-responsive regulatory elements located in untranslated regions of messenger RNAs (Serganov and Nudler 2013). They change their conformation in response to specific metabolite binding (Roth and Breaker 2009; Edwards and Batey 2010; Serganov and Patel 2012), and they have been proposed as modern descendants of an ancient sensory and regulatory system in the RNA world (Breaker 2012). Many pathogenic bacteria use riboswitches to control essential metabolic pathways, and they are currently regarded as promising antibacterial drug targets (Blount and Breaker 2006; Mulhbacher et al. 2010; Deigan and Ferré-D'Amaré 2011). Riboswitches consist of an aptamer domain that binds the effector ligand and an expression platform that transduces the ligand-induced conformational “switch” into a modulation of gene expression (Barrick and Breaker 2007; Garst and Batey 2009). Among more than 20 natural aptamer classes (Breaker 2012), purine-sensing riboswitches have the peculiarity to recognize the targeted purine by utilizing a conserved pyrimidine (Kim and Breaker 2008; Batey 2012). One of the most characterized members of this class is the adenine sensing riboswitch (A-riboswitch) *cis*-regulating the *add* gene in *Vibrio vulnificus* (Mandal and Breaker 2003). The ligand-bound structure of its aptamer is a three-way junction composed of three stems (P1, P2, P3) with the ligand completely encapsulated into the structure (Fig. 1; Mandal and Breaker 2004; Serganov et al. 2004). The specificity for adenine is ensured by canonical Watson-Crick (WC) base-pairing established between a uracil in the conserved position and the ligand (Noeske et al. 2005; Gilbert et al. 2006).

**FIGURE 2.**
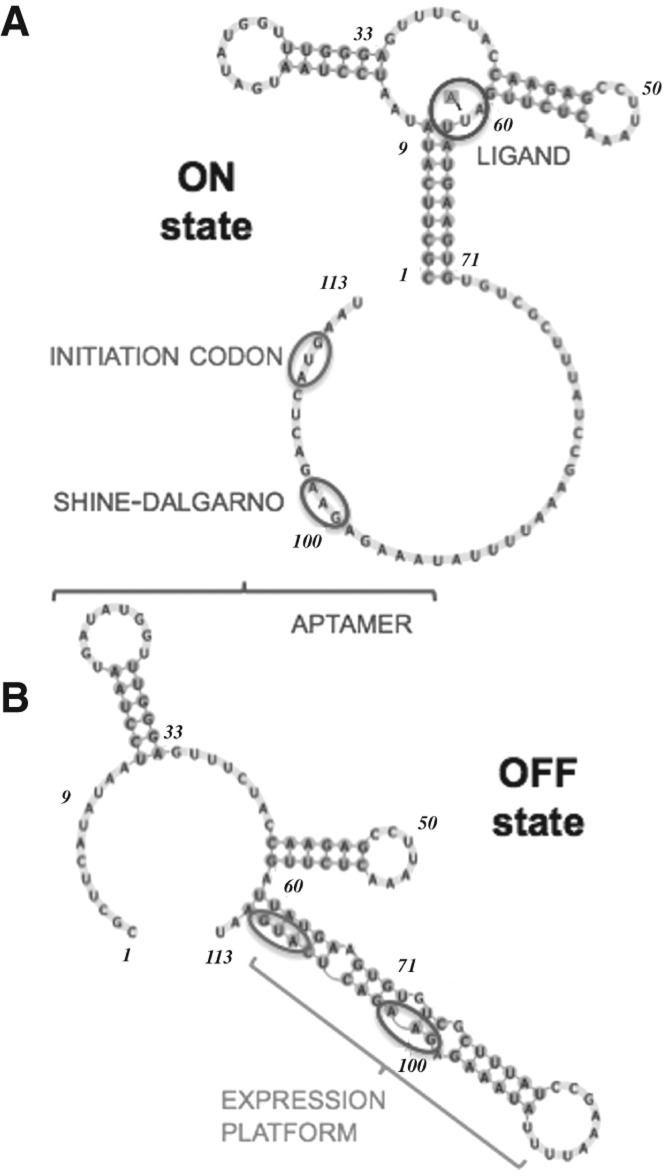
Secondary structure representation of the *add* riboswitch in the ON-state (*A*) and OFF-state (*B*). The ligand, the initiation codon, and the Shine-Dalgarno sequence are labeled.

The A-riboswitch acts as a translational regulator (Serganov et al. 2004; Lemay et al. 2011). In the absence of adenine, the ribosome binding site and the initiation codon, which are portions of the expression platform, are sequestered by pairing with a portion of the aptamer (OFF-state) (Fig. 2B). The presence of adenine stabilizes an aptamer conformation in which the terminal P1 helix is well structured, and both the regulatory sequences are available for ribosomal binding, thus enabling mRNA translation (ON-state) (Fig. 2A; Rieder et al. 2007; Lee et al. 2010; Leipply and Draper 2011). The structural mechanism regulating the switch between the ON- and the OFF-state upon ligand binding mostly remains to be elucidated. The P1 stem is formed in the ON-state and disrupted in the OFF-state (Mandal and Breaker 2003; Serganov et al. 2004).

It has been proposed that P1 is stabilized by the ligand (Batey et al. 2004), and this could be a common feature in many riboswitch classes (Montange and Batey 2006). The role of ligand binding in the structural organization of the aptamer has been investigated with single-molecule spectroscopy, providing an insightful overview on the folding dynamics (Neupane et al. 2011), yet lacking the critical atomistic details needed for an accurate structural characterization of the process as extensively discussed by Lin et al. (2012). Although in silico techniques have been used to investigate the ligand role (Lin and Thirumalai 2008; Sharma et al. 2009; Priyakumar and MacKerell 2010; Gong et al. 2011; Allnér et al. 2013), a quantitative estimation of the energetic contributions associated to ligand binding, in particular regarding the role of direct P1-ligand interactions, has not yet been provided. In this context, state-of-the-art free-energy methods combined with atomistic simulations can bridge the gap, providing an unparalleled perspective on the mechanism and dynamics of the biomolecular process of interest (Dellago and Bolhuis 2009). In this work, we used steered molecular dynamics (SMD) (Grubmüller et al. 1996; Sotomayor and Schulten 2007) simulations to study the thermodynamics of the P1 stem formation in the presence and in the absence of the cognate ligand. We enforced the breaking of the P1 stem base pairs (bp), and then using a recently developed reweighting scheme (Colizzi and Bussi 2012), we quantitatively estimated the ligand-induced stabilization of the helix. The A9-U63 bp, which directly stacks with adenine, was used as a proxy for the P1 stability. Our nonequilibrium simulations provide measurements of the stability of the A9-U63 bp and quantify the direct ligand-dependent stabilization of the pairing. In the following, our results are presented and compared with melting and single-molecule experiments. A structural model for the conformational switch emerging from the combination of our results and previous experimental data is also discussed.

## RESULTS

**FIGURE 3.**
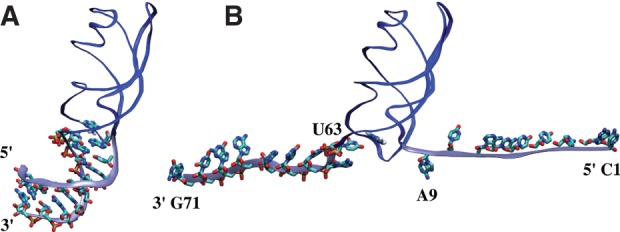
Initial (*A*) and final (*B*) configuration of the SMD simulation opening the P1 stem shown here for the Holo form. The backbone of the aptamer is in blue except for the P1 stem, which is in light blue. The ligand and the 18 bases forming the helix are shown. The P1 stem is formed in *A* and disrupted in *B*.

**FIGURE 4.**
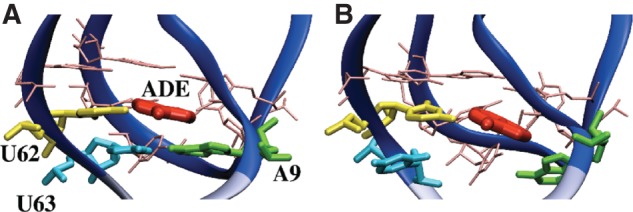
Representative structures of the Holo binding pocket at the beginning (RMSD = 0 nm) (*A*) and at the end (RMSD = 0.35 nm) (*B*) of the SMD. The portion of the P1 stem removed in our simulations is in light blue. Bases forming the binding pocket are labeled, ligand is shown in red. A9-U63 pair is formed in *A* and disrupted in *B*.

We carried out the simulations of the aptamer domain of the *add* A-riboswitch in different forms, namely, the entire aptamer (PDB id 1Y26) (Serganov et al. 2004) has been simulated in the presence (Holo) and in the absence (Apo) of the cognate ligand, the adenine; additionally, to better estimate the ligand-induced stabilization, we also simulated a truncated aptamer (Δ1–8/64–71), both in the Apo and Holo forms. Long unbiased molecular dynamics (MD) for all four systems were performed to test the stability of the aptamer in different conditions. In the truncated systems, the terminal bp was restrained in its initial configuration to mimic the presence of the rest of the stem. Furthermore, the full-length systems were pulled from the terminal bases to disrupt the entire P1 stem (Fig. 3), thus allowing its different stability between the Holo and the Apo forms to be qualitatively inferred. At last, to quantify this difference, SMD simulations of both the Δ1–8/64–71 systems were done, enforcing the breaking of the A9-U63 bp that directly stacks with the ligand (Fig. 4).

**FIGURE 5.**
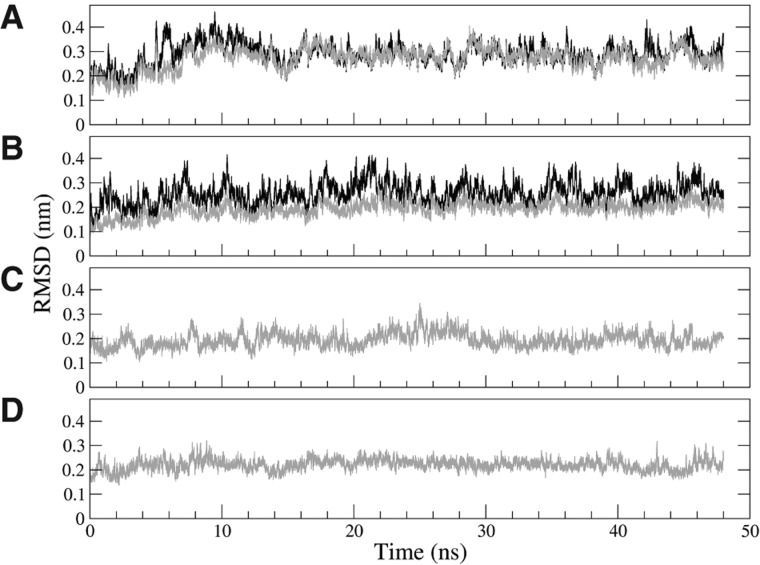
RMSD from native structure. (*A*) Holo form during 48-nsec equilibration, computed on the whole aptamer (black) and on the bases from 9 to 63 (gray). (*B*) Same as *A* done on the Apo form (whole aptamer, black; bases from 9 to 63, gray). The difference between black and gray profiles, in both panels, indicates that the P1 stem is less stable than the rest of the aptamer. (*C*) Δ1–8/64–71 Holo RMSD along the 48-nsec equilibration at constant temperature. (*D*) Same as *C* for the Δ1–8/64–71 Apo form.

The stability of both the Apo and Holo systems was evaluated monitoring the root mean square deviation (RMSD) from the native structure along 48-nsec MD runs (Fig. 5A,B). Ligand removal (see Materials and Methods for details) did not affect the overall stability of the Apo aptamer in this time-scale, and secondary and tertiary structures were substantially unchanged.

**FIGURE 6.**
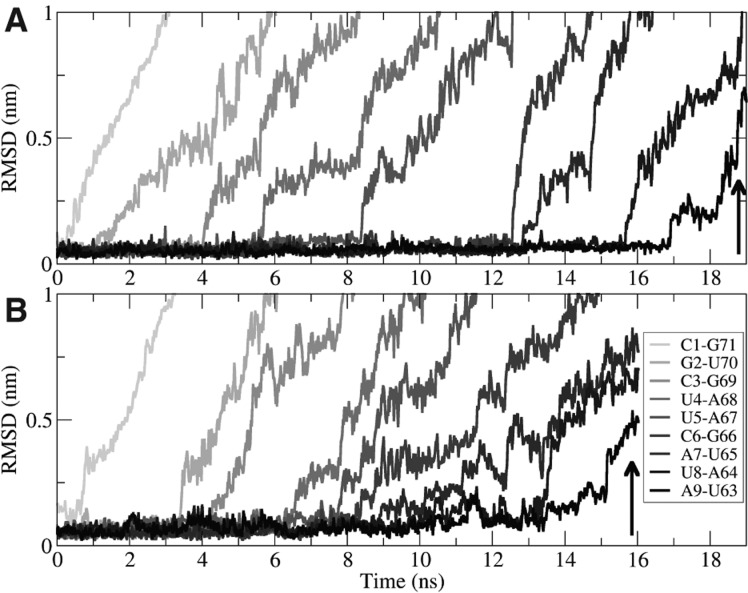
Base-pair ruptures during P1 pulling. In the pulling simulations, the 9 bp forming the P1 stem were unpaired. Here, we monitored the RMSD of each bp (gray scale) from their native conformation: (*A*) Holo; (*B*) Apo. A9-U63 bp (in black) was disrupted (RMSD ≈0.5 nm, arrows) later in the Holo form (≈19 nsec) than in the Apo form (≈16 nsec).

The analysis of the trajectories obtained by pulling the P1 stem showed that the secondary and tertiary structure elements of the rest of the aptamer were not affected by the opening of the helix (data not shown). Focusing our attention on the P1 stem, we observed that in the Apo form, the A9-U63 bp (Fig. 3) was broken when the distance between the centers of mass of the terminal bases reached a value of ≈9.8 nm. In contrast, in the presence of the ligand a longer pulling was needed, and the rupture only happened at a distance of ≈11.5 nm (Fig. 6). This behavior is compatible with the picture in which the ligand stabilizes the P1 stem (Montange and Batey 2006). It was, however, difficult to extract quantitative information on the ligand–P1 interaction from these simulations because the rupture is a stochastic event, and extensive sampling would be required. Moreover, as pointed out in a recent paper (Lin et al. 2012), the end-to-end distance could be a nonoptimal collective variable (CV) for pulling experiments or simulations since local bp formation plays an important role in global stem folding.

The quantitative analysis of the P1–ligand interaction was better obtained from the simulation of both the Δ1–8/64–71 systems. We verified that when the P1 stem is replaced with the A9-U63 bp restrained to be in canonical WC pairing, the aptamer remains stable (Fig. 5C,D). Remarkably, fluorescence experiments have shown that the aptamer can also fold and bind adenine when large fractions of the P1 stem are removed (Lemay and Lafontaine 2007). This validates the possibility of using the two structures, Δ1–8/64–71 Holo and Δ1–8/64–71 Apo, to investigate the direct P1 stabilization given by the adenine. In the following, we focus on the SMD simulations performed on these truncated forms. Typical initial and final conformations from the SMD are shown in Figure 4.

### Analysis of work profiles

**FIGURE 7.**
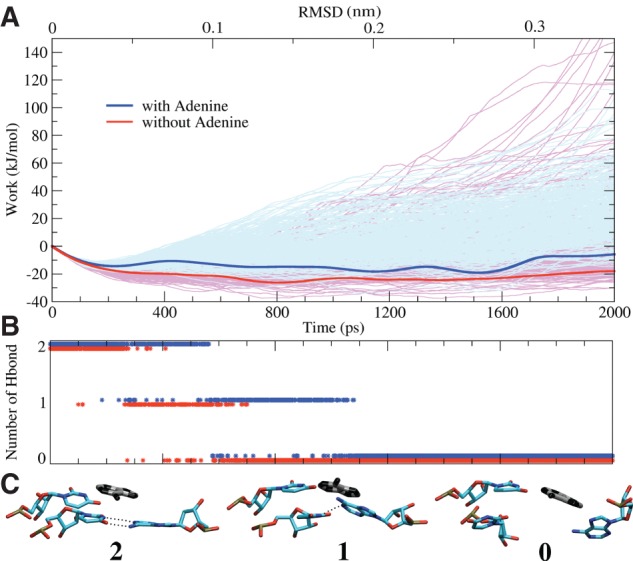
Unbinding process of the A9-U63 bp. (*A*) Mechanical work performed as a function of the value of the steered RMSD, or equivalently of time, for 512 simulations for Apo (pink) and Holo form (light blue). The respective free energies resulting from the Jarzynski equality (Jarzynski 1997a) are shown in thicker red and blue lines. The initial free-energy decrease related to the entropy gain induced by the restraint movement has no consequence on the final result. (*B*) Hydrogen bonds occurrence for both the systems Apo (red) and Holo (blue). (*C*) Snapshots of the Holo form (ligand in black) with two, one, or zero hydrogen bonds (dotted lines) formed between A9 and U63.

The unbinding event of the A9-U63 bp is described as a function of the value of the steered RMSD in Figure 7. The initial value corresponds to the configuration with the WC pairing formed, whereas at the final value (0.35 nm) the pairing is completely broken. Even if the ensembles of work profiles for the two forms are broadly spread and superposed, the free-energy profiles, computed using the Jarzynski equality (Jarzynski 1997a) as the exponential average of the two sets of data, are clearly distinguishable (Fig. 7A). Qualitatively it is worth highlighting that during the breaking of the A9-U63 bp, the Apo form (red line) is always lower in free energy than the Holo form (blue line). It follows that the breaking of the monitored bp in the Apo form was unambiguously more probable than in the Holo one (Fig. 7A). However, such an approach was still a long way off from quantitatively accounting for the energetic stabilization of the A9-U63 bp related to the presence of adenine in the binding site. Within this framework, there was no way to automatically detect when the nucleobases reached the unbound configuration. It was thus difficult to avoid systematic errors in the comparison of the two systems. Furthermore, few low-work realizations occurred during the unpairing in the presence of adenine. In these low-work realizations, the number of hydrogen bonds was nonzero at large RMSD values, and the structural analysis of the trajectories revealed the transient formation of a *cis*-sugar edge pair (data not shown) (Leontis et al. 2002). Due to the exponential nature of the Jarzynski average (Jarzynski 1997a), these low-work realizations dominated the free energy profile for the Holo form, further compromising the possibility of a quantitative comparison with the Apo form.

### Energetics of hydrogen-bond breaking

We thus analyzed the trajectories in terms of number of hydrogen bonds formed between A9 and U63, a discrete variable that more strictly reported on the breaking of the pairing. In this metric, the bound (one or two hydrogen bonds) and unbound (zero hydrogen bond) ensembles could be clearly and unambiguously identified, thus allowing a quantitative comparison between the Apo and the Holo system. Additionally, the configurations from the outlier trajectories could be assigned properly to one or the other ensemble in spite of their atypical RMSD value.

The differences in free energy (Δ*F*) between the ensembles, with and without hydrogen bonds, was computed using a reweighting scheme (Colizzi and Bussi 2012). The values and the associated standard errors were estimated for both systems: For the Apo form, Δ*F*= −2.5 ± 1.4 kJ/mol, suggesting that the bp could spontaneously break in the absence of adenine; and for the Holo form, Δ*F*= 1.9 ± 1.7 kJ/mol, implying that the presence of the ligand and its pairing with U63 stabilized the stacked bp. The ΔΔ*F* between the two forms is equal to −4.4 ± 2 kJ/mol. This value quantifies the thermodynamic stabilization to the formation of the base pair, which directly interacts with adenine in the P1 stem.

## DISCUSSION

Our simulations at atomistic detail provide for the first time the free-energy contribution of ligand stacking to the formation of the P1 stem in a riboswitch. In particular, the presented in silico approach allows the energetics involved in the aptamer stabilization upon ligand binding to be dissected in detail. Below, we compare our results with single-molecule manipulation, both in vitro and in silico, and thermodynamic data from dsRNA melting experiments. We also provide a model for ligand-modulated cotranscriptional folding of the *add* riboswitch.

### Comparison with related works

Our results are in nice agreement with thermodynamic data based on dsRNA melting experiments (Mathews et al. 2004; Turner and Mathews 2010). The comparison between our simulations and those experiments can be straightforwardly achieved by considering the pairing between U62 and the sensed adenine as an additional terminal bp of the P1 stem. The direct stabilization of the P1 stem due to the cognate-ligand binding should be thus equivalent to that given by adding one further AU bp to the P1 helix. Using the most recent nearest-neighbor energy parameters for the comparison of RNA secondary structures (Mathews et al. 2004; Turner and Mathews 2010), the free-energy difference between the sequence of the P1 stem with and without the additional AU bp,

5′-CGCUUCAUA*A*-3′
3′-GUGAAGUAU*U*-5′

and

5′-CGCUUCAUA-3′
3′-GUGAAGUAU-5′

can be computed (Hofacker et al. 1994; Lorenz et al. 2011) as ΔΔ*F*= −3.7 kJ/mol, consistently with our results.

**FIGURE 8.**
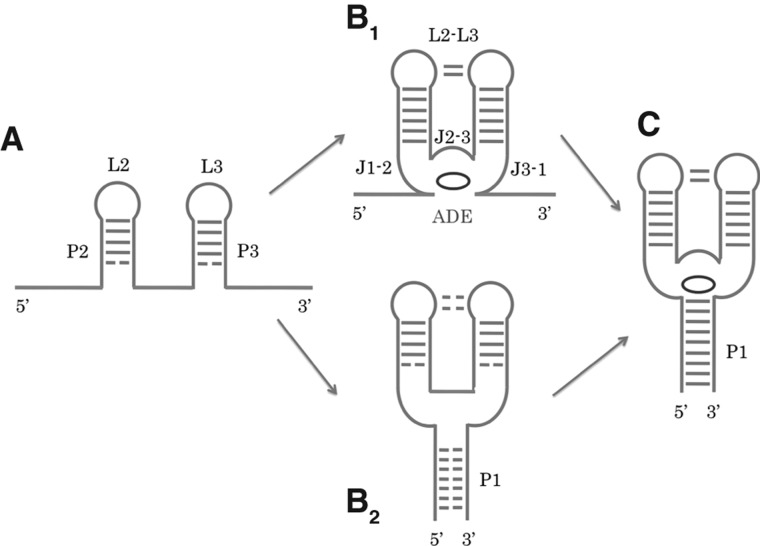
Schematic representation of the aptamer secondary structure in its folding intermediates with and without the ligand. (*A*) Stems P2 and P3, loops L2 and L3 are folded but not stable. (*B*_1_) The junctions (J1-2, J2-3, J3-1) arrange around the ligand (ADE) and the interloop pairings occur (L2–L3), stabilizing also the stems. (*B*_2_) Alternative possible intermediate in which all three stems are not completely and stably folded before ligand binding. The junctions and the tertiary interaction between the loops are not stable. (*C*) P1 stem is fully folded and stabilized by the ligand.

Our free-energy estimates complement previously reported investigations in which the role of the ligand in the folding process of the A-riboswitch has always been referred to the whole aptamer (Lin and Thirumalai 2008; Neupane et al. 2011) and never specifically to the P1 stem. Using a one-bead-per-nucleotide coarse-grained model, the ΔΔ*F* has been computed as approximately −15 kJ/mol (Lin and Thirumalai 2008). Notably, this calculation has also been done using a shortened P1 stem, possibly affecting the Δ*F* estimation. Single-molecule force spectroscopy experiments have been also performed to characterize the folding pathway of the aptamer with an estimated ΔΔ*F* ≃ −33 kJ/mol (Neupane et al. 2011). However, in both works, the separated contributions of the P1-ligand stacking, of the interaction between the ligand and the junctions J1-2, J2-3, and J3-1, and of the interaction between loops L2 and L3, could not be discerned (secondary structure elements labeled as in Fig. 8).

From the comparison of our data with the aforementioned experimental and computational works, a twofold modular role for the ligand emerges. On the one hand, the binding of adenine can contribute to the aptamer preorganization and could allow the long-range induction of the tight hydrogen-bonding and base-stacking networks observed in the native state (Rieder et al. 2007; Lee et al. 2010). This preorganization would reduce the distance between A9 and U63, thus increasing the probability of their pairing. A similar mechanism has been proposed also for the SAM-I riboswitch (Whitford et al. 2009). On the other hand, adenine binding enhances the P1 formation by direct stacking interaction, mimicking the extension of the helix by an additional bp. Notably, the energetic contribution of the direct stacking is smaller than that involved in the aptamer preorganization. The latter can be estimated as the difference between the global ligand-induced aptamer stabilization (Lin and Thirumalai 2008; Neupane et al. 2011) and the stacking contribution dissected in our work.

### Folding model

Our work provides atomistic details and energetic estimates to the currently accepted model for the folding of the *add* riboswitch upon ligand binding (Rieder et al. 2007; Lee et al. 2010; Leipply and Draper 2011). Altogether, our data and the related experimental works suggest a folding model as depicted in Figure 8. Initially, only the P2 and P3 stems and the corresponding loops (L2, L3, still not interacting with each other) are formed and not fully stable (Fig. 8A). Then, adenine binding allows for a preorganization of the aptamer, where the three junctions arrange around the ligand (Fig. 8B_1_), stabilizing also the previously formed helices (Rieder et al. 2007). It has not been clearly established if the loop–loop interaction is formed before or after ligand binding (Leipply and Draper 2011). Thus, in an alternative pathway, the junctions and the P1 could acquire a partially folded conformation also in the absence of adenine (Fig. 8B_2_; Lee et al. 2010). Finally, the P1 helix becomes fully structured and stabilized by the ligand (Fig. 8C), to the detriment of the expression platform (see Fig. 2; Lemay et al. 2011). This step is mandatory for translation to be initiated. We quantified the ligand contribution to the P1 stem formation due to direct interactions to be approximately −4 kJ/mol.

Our result is compatible with both the folding pathways (Fig. 8B_1_,B_2_) irrespective of their relative population and cannot discriminate among them. The relative probability between the two paths can be modulated by the ligand concentration and its binding affinity. On the one hand, the intermediate shown in Figure 8B_1_ could be relevant for ligand–RNA binding in an early transcriptional context in which the last nine nucleobases (i.e., those allowing P1 formation) of the aptamer have not yet been synthesized. Indeed, it has also been shown that an aptamer missing a large portion of the P1 stem is able to bind adenine (Lemay and Lafontaine 2007). On the other hand, the intermediate shown in Figure 8B_2_ could be populated at low-ligand concentration once the nucleobases allowing P1 formation are synthesized. Later on, after the synthesis of the expression platform, ligand binding could shift the thermodynamic equilibrium toward one of two competing riboswitch conformations (P1 formed and nonformed).

### Conclusion

Ligand-induced stabilization of the P1 stem is crucial for A-riboswitch regulation and function. Here, we quantified the direct interaction between adenine and the P1 stem and analyzed it at atomistic detail. Our results suggest a model for the aptamer folding in which the direct P1-ligand interactions play a minor role on the conformational switch when compared with those related to the ligand-induced aptamer preorganization. Because the structural/functional role of the aptamer terminal helix is a common feature in the “straight junctional” riboswitches (Serganov and Nudler 2013), we foresee a wider validity of the model presented herein.

## MATERIALS AND METHODS

### System description and set-up

We simulated the Holo and the Apo form of the A-riboswitch aptamer domain both composed of 71 nucleotides. The Apo form was generated by adenine removal from the ligand-bound (Holo) crystal structure (PDB id: 1Y26) (Serganov et al. 2004). This deletion is justified by the fact that the Apo and Holo form have been shown experimentally to share an overall similar secondary structure (Lemay et al. 2011). This is at variance with, for example, the *pbuE* adenine riboswitch in which the two structures are different. The generation of the Apo form by simply removing the ligand has been adopted also in a recent work (Allnér et al. 2013). Molecular dynamics (MD) simulations were performed using the Amber99 force field (Wang et al. 2000) refined with the *parmbsc0* corrections (Pérez et al. 2007). From the analysis of the SMD trajectories, we do not expect the refinement on the χ torsional parameters (Zgarbová et al. 2011) to affect the results. Adenine was parameterized using the general Amber force field (gaff) (Wang et al. 2004). Partial atomic charges were assigned using the restricted electrostatic potential fit method (Bayly et al. 1993) based on an electronic structure calculation at the HF/6-31G* level of theory performed with Gaussian03 (Frisch et al. 2004). Bond-lengths were constrained with LINCS (Hess et al. 1997), and the electrostatic interactions were calculated using the particle-mesh Ewald method (Darden et al. 1993). For both forms, the following protocol was used to prepare the systems (Table 1 for details) for MD simulations: steepest descent minimization (200 steps) starting from the X-ray structure; solvation with ≈13,000 TIP3P water molecules (Jorgensen et al. 1983) and NaCl at 0.15 M concentration (plus extra Na^+^ counter-ions to neutralize the charges of the systems) in a hexagonal prism (lattice vectors in nm [(10,0,0), (0,7,0), (0, 7/2, 
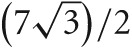
)]) that was created orienting the major length of the aptamer along the *x*-axis; steepest descent minimization (200 steps) for ions and solvent; the systems were thermalized at 300 K, initially for 200 psec with frozen solute positions and then for 5 nsec in NPT ensemble (1 atm) with stochastic velocity rescaling (Bussi et al. 2007) and Berendsen barostat (Berendsen et al. 1984); to maintain the systems oriented along the largest lattice vector (X) a restraint was imposed with a force constant of 4 ⋅ 10^3^(kJ/mol)/nm^2^ on the Y and Z components of the distance between phosphate atoms of A52 and G71. Each system was simulated for 48 nsec in NVT ensemble to assess the stability of the aptamer.

**TABLE 1.**
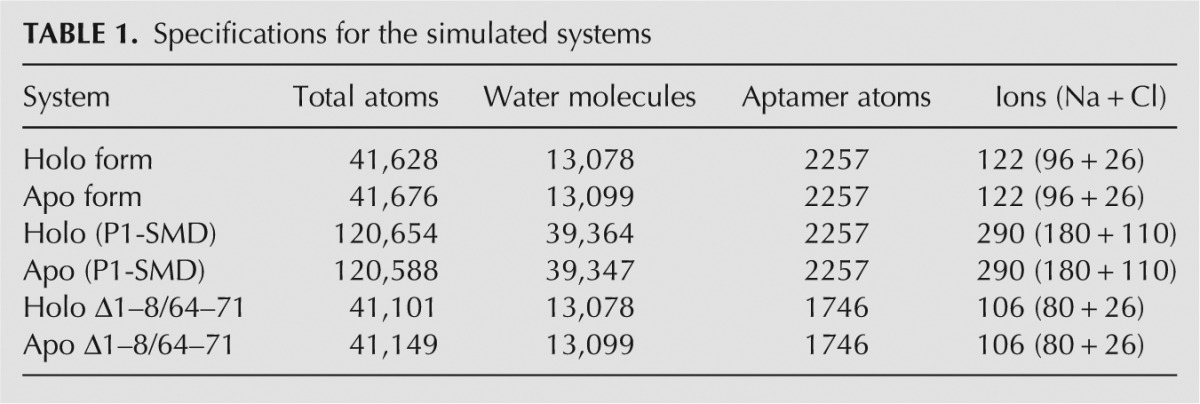
Specifications for the simulated systems

### Steered molecular dynamics

To perform SMD simulations inducing the opening of the whole P1 stem, the systems were solvated again with ≈39,500 water molecules in a larger rhombic dodecahedral box with distance between periodic images equal to 12 nm, adding ions to maintain the same ionic strength (P1-SMD systems in Table 1). The same protocol described above was applied for the minimization, thermalization, and equilibration of this larger Holo and Apo systems for the pulling simulations. An incremental separation between the centers of mass of the terminal nucleotides (C1 and G71) was imposed from an initial value of 1.05 nm to a value sufficient to completely unfold the 9 bp of the P1 helix (Apo 10.05 nm, Holo 11.75 nm) at a speed of 0.56 nm/nsec (see Fig. 3). The spring constant was set to 3.9 ⋅ 10^4^(kJ/mol)/nm^2^.

The first eight bp of the P1 stem (i.e., whole stem from C1 to U8 and from A64 to G71, except for A9-U63 bp) were then cut in both systems, creating the Δ1–8/64–71 Holo and Δ1–8/64–71 Apo structures (Table 1 for details). Water molecules were allowed to relax, filling the space left by the 16 removed bases through an additional 1-nsec equilibration in which the positions of aptamer atoms were frozen followed by 5 nsec of unrestrained NPT simulation. Then the systems were simulated for 48 nsec in the NVT ensemble, restraining the terminal bases in the initial state to avoid any spontaneous flipping. The pairs deletion is not biologically meaningless because it has been shown experimentally that a series of aptamer variants with shorter P1 helix are still able to bind the ligand (Lemay and Lafontaine 2007). The deletion reduced the noise during the pulling, allowing the calculation to be focused on the influence of the ligand on the A9-U63 pairing. This bp rupture was here enforced by pulling on the RMSD between the heavy atoms of A9 and U63 with reference to the crystal structure. This CV was chosen as it identifies the native conformation (RMSD ≈ 0) of the A9-U63 bp, which is necessary for the initiation of the P1 stem formation. The steered CV was pulled at constant velocity of 0.175 nm/nsec from 0 to 0.35 nm in 2 nsec. This pulling induced the complete opening of the A9-U63 bp in the presence and absence of the ligand (Fig. 4). The spring constant was set to 3.9 ⋅ 10^4^(kJ/mol)/nm^2^. The starting points were extracted equidistantly (one every 16 psec) from a 8.192-nsec run NVT ensemble restraining the RMSD value of those atoms at 0. For the two systems, 512 independent SMD simulations were performed, corresponding to an aggregate time of ∼1 μsec each. Simulations were carried out with the Gromacs 4.0.7 program package (Hess et al. 2008) combined with the PLUMED 1.3 plug-in (Bonomi et al. 2009).

### Analysis

The Jarzynski equality (Jarzynski 1997a) was used to estimate the equilibrium free-energy landscape of the process from the collected work profiles. The simulations were then analyzed using a recently proposed scheme (Colizzi and Bussi 2012), which combines an identity by Jarzynski (Jarzynski 1997b) with the weighted-histogram analysis method (Kumar et al. 1992). The algorithm allows the free-energy profiles to be projected onto any a posteriori chosen CV. It is well known that free-energy calculations using Jarzynski-based relationships are difficult to converge. Statistical errors were thus estimated by the bootstrapping procedure described in Do et al. (2013), indicating that our results were converged within ≈*k_B_T*. The VIENNA RNA package (Hofacker et al. 1994; Lorenz et al. 2011) was used to compare our results with the thermodynamic data based on dsRNA melting experiments (Mathews et al. 2004; Turner and Mathews 2010).
